# Epicutaneous immunization with modified vaccinia Ankara viral vectors generates superior T cell immunity against a respiratory viral challenge

**DOI:** 10.1038/s41541-020-00265-5

**Published:** 2021-01-04

**Authors:** Youdong Pan, Luzheng Liu, Tian Tian, Jingxia Zhao, Chang Ook Park, Serena Y. Lofftus, Claire A. Stingley, Yu Yan, Shenglin Mei, Xing Liu, Thomas S. Kupper

**Affiliations:** 1 Department of Dermatology and Harvard Skin Disease Research Center, Brigham and Women’s Hospital, Harvard Medical School, Boston, Massachusetts USA; 2 Beijing Institute of Traditional Chinese Medicine, Beijing Hospital of Traditional Chinese Medicine, Capital Medical University, Beijing, China; 3 Department of Biomedical Informatics, Harvard Medical School, Boston, Massachusetts USA; 4 The Center for Microbes, Development and Health, Key Laboratory of Molecular Virology and Immunology, Institut Pasteur of Shanghai, Chinese Academy of Sciences, Shanghai, China; 5 Dana-Farber/Brigham and Women’s Cancer Center, Boston, Massachusetts USA

**Keywords:** Immunology, Infectious diseases

## Abstract

Modified Vaccinia Ankara (MVA) was recently approved as a smallpox vaccine. Variola is transmitted by respiratory droplets and MVA immunization by skin scarification (s.s.) protected mice far more effectively against lethal respiratory challenge with vaccinia virus (VACV) than any other route of delivery, and at lower doses. Comparisons of s.s. with intradermal, subcutaneous, or intramuscular routes showed that MVA_OVA_ s.s.-generated T cells were both more abundant and transcriptionally unique. MVA_OVA_ s.s. produced greater numbers of lung Ova-specific CD8^+^ T_RM_ and was superior in protecting mice against lethal VACV_OVA_ respiratory challenge. Nearly as many lung T_RM_ were generated with MVA_OVA_ s.s. immunization compared to intra-tracheal immunization with MVA_OVA_ and both routes vaccination protected mice against lethal pulmonary challenge with VACV_OVA_. Strikingly, MVA_OVA_ s.s.-generated effector T cells exhibited overlapping gene transcriptional profiles to those generated via intra-tracheal immunization. Overall, our data suggest that heterologous MVA vectors immunized via s.s. are uniquely well-suited as vaccine vectors for respiratory pathogens, which may be relevant to COVID-19. In addition, MVA delivered via s.s. could represent a more effective dose-sparing smallpox vaccine.

## Introduction

Vaccines against viral and bacterial pathogens have become a fundamental part of pediatric and adult patient care^1–4^. Once ubiquitous diseases such as smallpox, polio, measles, tetanus, and diphtheria have either been eliminated or substantially reduced in incidence by vaccination in most of the industrialized world. Vaccination against seasonal influenza has been more challenging, and vaccination against human immunodeficiency virus has proven elusive^5–7^. Vaccines against emerging diseases such as Ebola, severe acute respiratory syndrome, and Middle East respiratory syndrome, and most recently Coronavirus disease 2019 (COVID-19) are the subject of intense interest and widespread activity^8–10^. Most vaccines are administered by intramuscular (i.m.) or subcutaneous (s.c.) injection. Although readily accessible, skeletal muscle and s.c. adipose tissues are poorly adapted to initiating immune responses^11^. In contrast, upper layers of the skin are the site of continuous and multiple immune responses over a lifetime^12^. Smallpox vaccination through epidermis with vaccinia virus (VACV) has been uniquely successful^2,11^.

The eradication of smallpox by worldwide epicutaneous immunization (skin scarification, s.s.) with VACV was arguably the greatest public health achievement of the twentieth century^2^. Since that time, VACV has been employed as a vaccine vector in many settings^13^. However, its use has been limited by unacceptable morbidity, particularly in recipients who are immunocompromised^14^. More recently, Modified Vaccinia Ankara (MVA), a replication-deficient variant of VACV, has come into wider use^15^. Although it lacks ~10% of the parent genome^16^, it retains the immunogenicity of the parent virus and has just been approved by the Food and Drug Association as a modern alternative for preventative smallpox vaccination^17–19^. Similar to VACV, it is also being widely used as heterologous vaccine vector^20^. However, MVA and derivative vectors are almost invariably delivered i.m. or s.c.^19,21^.

Several important features of smallpox vaccination deserve to be re-emphasized. Development of a cutaneous “pox” lesions, achieved only after s.s. immunization, was considered emblematic of successful protective vaccination, suggesting that this mode of delivery was critically important^14^. In addition, smallpox vaccination with VACV was effective in patients with agammagloblulinema but had disastrous complications in patients with T-cell deficiency^22^. This suggested that T cells were critically important for protective immunity^23,24^. Finally, Variola virus is transmitted via respiratory droplets, suggesting an oropharyngeal-pulmonary mode of transmission^25^. It is notable that murine models of epicutaneous skin immunization with VACV generate memory T-cell populations in both the skin and lung, and these lung memory T cells protect against lethal pulmonary challenge with this virus^11^. Immunization (i.m.) with VACV in these models did not yield comparable protection. This suggests that protection against smallpox is at least in part mediated by T cells^24,26^, and that skin immunization is an effective means of generating protective memory T-cell populations in the lung^11^.

Given the unacceptable morbidity of VACV in humans, in this study, we aimed to assess the safety and efficacy of MVA vaccination, and asked whether s.s. immunization with MVA was superior to other routes of administration. We also asked which populations of skin antigen-presenting cells play a major role during MVA s.s. vaccination-induced protection. In addition, we asked whether skin immunization with an MVA vector encoding a CD8^+^ T-cell antigen could generate a population of antigen-specific CD8^+^ T cells in the lung and skin. Finally, we studied the early imprinting of activated CD8^+^ T cells in lymph nodes (LNs) draining the skin, lung, and gut after vaccination with MVA encoding a CD8^+^ T-cell antigen.

## Results

### MVA immunization via s.s. elicits dose-dependent anti-vaccinia immune response

**Fig. 1:**
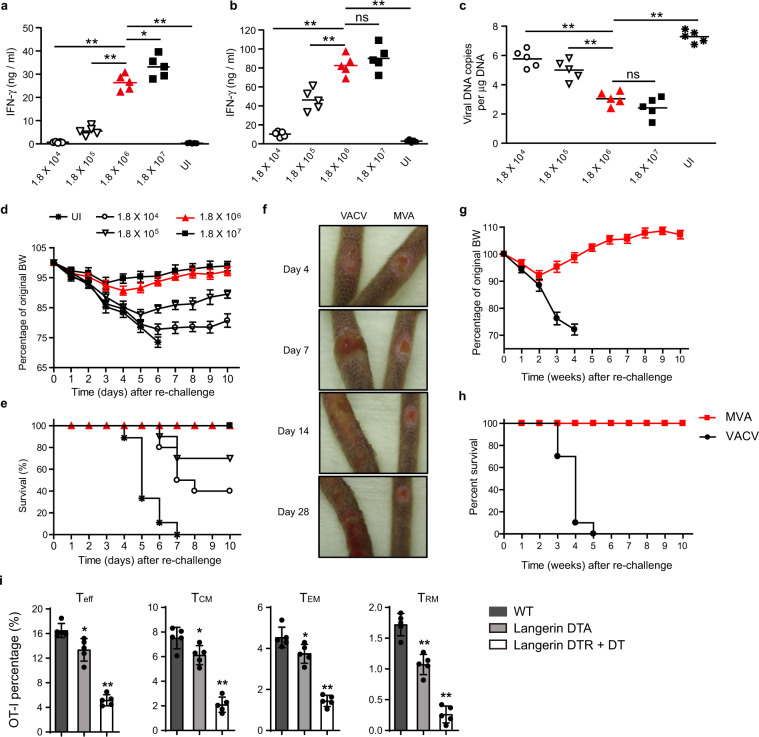
MVA immunization via skin scarification (s.s.) elicits dose-dependent anti-VACV immune response. **a**, **b** IFN-γ secretion by vaccinia-specific T cells isolated from draining lymph nodes (**a**) or spleens (**b**) at 7 days post MVA infection at indicated dose. **c** Quantitative real-time PCR (qRT-PCR) analysis of skin viral load at 6 days post re-infection. Mice were immunized with the indicated doses of MVA via s.s. 45 days later, mice were re-challenged with 1.8 × 10^6^ pfu vaccinia virus (VACV). Then 6 days later, skin tissues were collected and processed to qRT-PCR. **d**, **e** Body weight (BW) (**d**) and survival measurements (**e**) of WR-VACV re-challenged mice that were immunized previously with MVA at indicated dose 45 days earlier. **f** Photographs of pox lesion in Rag1^−/−^ mice taken on day 4, 7, 14, and 28 post immunization with the same amount (1.8 × 10^6^ pfu) of MVA or VACV. **g**, **h** Immunized Rag1^−/−^ mice were monitored for BW change (**g**) and survival (**h**) for up to 12 weeks after immunization with the same amount (1.8 × 10^6^ pfu) of MVA or VACV. **i** Quantification of effector T cell (T_eff_, day 5), central memory (T_CM_, day 45), effector memory (T_EM_, day 45), or tissue resident memory (T_RM_, day 45) T cells post MVA infection. Naive OT-I Thy1.1^+^ cells were transferred into Thy1.2^+^ recipient mice 1 day before mice were infected with 1.8 × 10^6^ pfu MVA-Ova. Then, at different time points post infection, OT-I cells were isolated from the lymph nodes (T_eff_, T_CM_, T_EM_) or skin (T_RM_) and analyzed by flow cytometry. **a**–**c** Data are representative of three independent experiments. Symbols represent individual mice (*n* = 5 mice/group). **a**–**d** Unimmunized (UI) mice were included as controls. Graphs show mean ± SD (*n* = 5), ns = not significant, *p < 0.05, **p < 0.01.

Doses from 10^4^ to 10^7^ pfu of MVA were used for s.s. and, after 7 days, all T cells from the inguinal LNs or spleen were purified and stimulated in vitro with VACV-infected splenocytes, and interferon-γ (IFN-γ) production was measured. All MVA doses led to significant T-cell IFN-γ production, with 10^6^ and 10^7^ pfu being equally potent (Fig. 1a, b). Other groups of mice were immunized with these doses and after 30 days these mice were challenged on the skin with VACV. After 6 days, biopsies of the challenge sites were taken and VACV DNA was measured by PCR. All immunization doses led to diminished VACV DNA at the challenge site (compared to unimmunized controls), but 10^6^ and 10^7^ pfu immunization showed superior, albeit partial protection (Fig. 1c). Other groups of mice were immunized in an identical manner and were subjected to lethal intranasal infection with VACV at day 30. All unimmunized mice rapidly lost weight and succumbed to the infection. In contrast, 40% of 10^4^, 70% of 10^5^, and 100% of 10^6^ and 10^7^ pfu immunized mice survived the infection (Fig. 1d, e). Thus, 10^6^ pfu is the lowest MVA dose that provides both strong T-cell cytokine production, as well as optimal protective immunity against the skin and pulmonary infection.

### MVA could be safely inoculated to the skin of immunocompromised mice without morbidity

To test whether delivery of MVA to scarified skin could induce poxvirus-specific immune responses, we inoculated C57BL/6 mice with MVA or VACV by scarification. By 7 days after inoculation, a crusted lesion resembling a “pox” reaction had formed at the inoculation site in all the immunized mice. The pox lesions induced by MVA and VACV s.s. followed similar patterns of evolution (although with different size and kinetics), from macules to papules to vesicles and finally into pustules which ruptured and healed over time with scars (Supplementary Fig. 1). MVA-induced pox reactions did heal more rapidly than those induced by replication competent VACV (Supplementary Fig. 1). To determine the safety of MVA in immunocompromised hosts, we next s.s. immunized immunodeficient Rag1^−/−^ mice with VACV and MVA, respectively, and followed the mice for several weeks. Although both groups of mice lost some weight over the first 2 weeks, MVA immunized mice rapidly regained the weight and flourished over the next several weeks (Fig. 1g). In contrast, 100% of the VACV-immunized mice developed progressive weight loss and expanding cutaneous lesions of VACV infection, ultimately requiring killing (Fig. 1f–h). Thus, MVA can be administered safely to mice wholly deficient in adaptive immunity.

### Langerhans Cells (LC) and Langerin^+^ Dendritic Cells (DC) are required for generating optimal CD8^+^ T-cell responses

In another set of experiments, we s.s. immunized wild-type (WT) mice and mice deficient in either Langerhans cells (Langerin-DTA) or both Langerhans cells and langerin-positive dermal dendritic cells (Langerin-DTR + DT), respectively. Prior to infection, mice were loaded with OT-1 cells and the immunizing virus was MVA_OVA_. Spleen and LNs were collected at days 10 and 30, the skin was collected at day 30, and OT-1 T cells were counted. At day 10, effector T cells (T_eff_) cells were somewhat diminished in Langerin-DTA mice and more markedly diminished in Langerin-DTR + DT mice (Fig. 1i). At day 30, skin tissue resident memory T cells (T_RM_) were significantly diminished in Langerin-DTA mice and even more diminished in Langerin-DTR + DT mice (Fig. 1i). This pattern was also true for T cells bearing markers of central memory T cells (T_CM_) and effector memory T cells (T_EM_) in the LN and spleen. These data suggest that both LC and langerin-positive dermal DC play an additive role in optimal antigen presentation of MVA-encoded antigens to T cells.

### MVA s.s. generated T cells that were both more abundant and transcriptionally unique

**Fig. 2:**
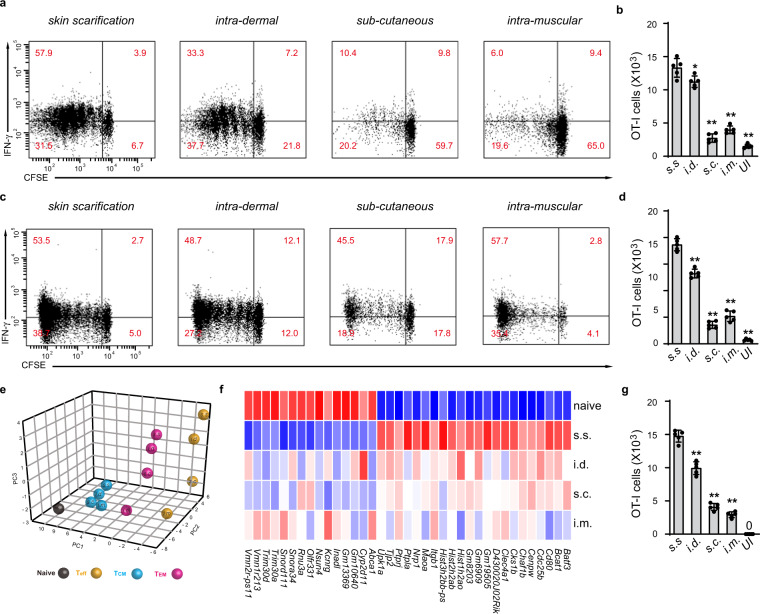
Delivery of MVA via s.s. generates T cells that are both quantitatively more abundant and qualitatively distinct from those generated from i.d., s.c., and i.m. **a**–**d** Flow cytometric analysis (**a**, **c**) and quantification (**b**, **d**) of OT-I cell proliferation in draining lymph nodes of recipient mice at 60 h (**a**, **b**) and 5 days (**c**, **d**) post MVA infection via different routes. CFSE-labeled naive OT-I Thy1.1^+^ cells were transferred into Thy1.2^+^ recipient mice 1 day before mice were infected with 1.8 × 10^6^ pfu MVA-Ova via indicated infection routes. **e** Principal component analysis (PCA) of gene expression for T cells generated by MVA infection via different routes. Naive T cells (T_N_) were sorted from the peripheral lymph nodes of naive OT-I mice. Effector T cells (T_eff_) were sorted from draining lymph nodes at 5 days post infection. Central memory T cells (T_CM_) and effector memory T cells (T_EM_) were sorted from the spleen of mice at 45 days post infection. Each dot represents an individual experiment wherein mRNA was pooled from 15 to 20 mice from 3 to 4 independent biological groups (5 mice/group). **f** Heatmap of differentially expressed genes selected from a pairwise comparison between s.s. generated T_eff_ cells and naive T cells. **g** Quantification of skin infiltrating T cells at day 5 post 1.8 × 10^6^ pfu MVA-Ova infection via indicated routes. **a**, **c** Data are representative of three independent experiments (*n* = 5 mice per group). **b**, **d**, **g** Unimmunized (UI) mice were included as controls. Graphs show mean ± SD (*n* = 5). *p < 0.05, **p < 0.01.

We next compared the anatomical route of vaccine delivery on the CD8^+^ T-cell response to MVA vaccination. Using carboxyfluorescein succinimidyl ester (CFSE) OT-1-loaded mice, MVA_OVA_ was delivered by s.s., or injected intradermally (i.d.), s.c., or i.m. Draining LNs were collected at 60 h and 5 days, and OT-1 cells were analyzed by fluorescence-activated cell sorting. LNs from s.s. immunized mice showed roughly 90% of OT-1 proliferating and 60% making IFN-γ, at 60 h, with comparable numbers at 5 days (Fig. 2a, c). Vaccination by i.d. was less effective, with 71% of OT-1 cells proliferating and 33% making IFN-γ at 60 h, with modest improvement at 5 days post infection (Fig. 2a, c). Both s.c. and i.m. showed poor OT-1 activation at 60 h with some improvement at 5 days (Fig. 2a, c). When LN or spleen OT-1 cells were stimulated with antigen, significantly more IFN-γ was produced by OT-1 cells from mice vaccinated via s.s. compared to other routes (Supplementary Fig. 2). Vaccination via i.d. was intermediate with regard to IFN-γ production, while s.c. and i.m. led to nearly fourfold lower IFN-γ levels (Supplementary Fig. 2). In terms of absolute numbers of OT-1 cells generated, s.s. was superior to all modes of vaccination, with i.d. being second and both i.m. and s.c. far less effective (Fig. 2b, d). We next took OT-1 cells from the 5-day post-immunization time point and performed transcriptional profiling on OT-1 cells generated after s.s., i.d., s.c., or i.m., respectively. Although there was some overlap, there were many differences between T cells generated by different routes of immunization, even at the same day post immunization (Fig. 2e and Supplementary Fig. 3). Principal component analysis (PCA) revealed that T_eff_ generated by s.s. and i.m. were transcriptionally distinct. T cells generated after s.s., i.d., and s.c., were more similar but still different from one another. T cells generated by s.s. and i.d. clustered closely but were still clearly not overlapping. Moreover, s.s generated most abundant skin infiltrating cells at day 5 post immunization (Fig. 2g).

### Delivery of MVA via s.s. generated more memory T cells and is superior in protecting mice against lethal respiratory challenge

**Fig. 3:**
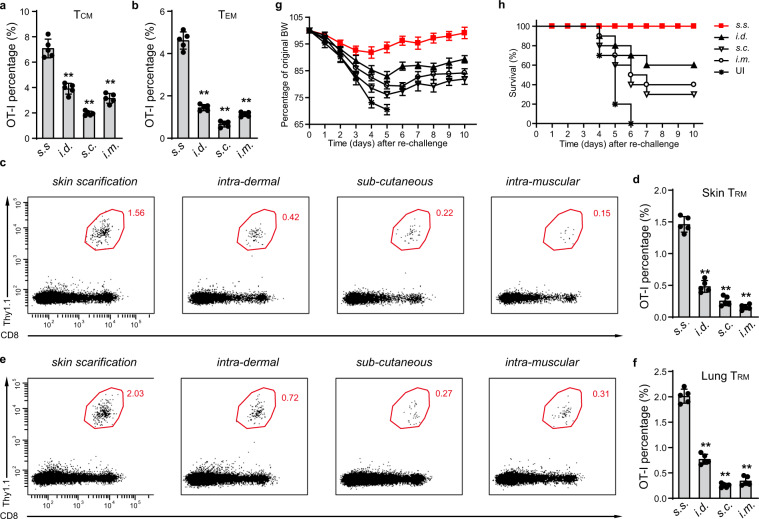
Delivery of MVA via s.s. is superior in generating memory T cells and is superior in protecting mice against lethal respiratory challenge. **a**, **b** Quantification of OT-I T_CM_ and T_EM_ cells from spleen of mice at 45 days post MVA infection via indicated routes. **c**–**f** Flow cytometric analysis (**c**, **e**) and quantification (**d**, **f**) of OT-I T_RM_ cells isolated from skin (**c**, **d**) or lung (**e**, **f**) tissue at 45 days post MVA infection via indicated routes. **g**, **h** Body weight (BW) (**g**) and survival measurements (**h**) of WR-VACV re-challenged mice that were previously immunized with MVA via indicated routes 45 days earlier. OT-I WT cells were adoptively transferred into µMT mice before mice were infected with 1.8 × 10^6^ pfu MVA via indicated routes. 45 days later, mice were re-challenged with a lethal dose of WR-VACV by intranasal infection. **c**, **e** Data are representative of three independent experiments (*n* = 5 mice per group). Graphs show the mean ± SD (*n* = 5). UI = unimmunized. **p < 0.01.

We next examined memory OT-1 T cells generated at 45 days by these four routes of immunization. With regard to T_CM_, s.s. generated the largest population of these cells, roughly twice as many as i.m. (Fig. 3a). The difference was even more striking when T_EM_ were examined; here, s.s. generated at least 3-fold more cells than did other modes of immunization, with s.c. being least effective (Fig. 3b). T_RM_ were then examined, in both skin and lung. Immunization via s.s. generated threefold more skin T_RM_ and more than twice as many T_EM_, with i.d. being the second most effective route (Fig. 3c–f). As MVA is often delivered i.m., it is important to note that the number of T_RM_ generated by this route was ~4-fold lower than by s.s. (Fig. 3c–f). Transcriptional profiling showed that at 45 days, OT-1 T_EM_’s still showed non-overlapping PCA clusters from s.s, i.d., s.c., and i.m. immunized mice. In contrast, T_CM_ from the same mice showed transcriptional profiles that were more tightly clustered, indicating that differences between the groups had become minimal (Fig. 2e). Skin T_RM_ could not be compared because insufficient 45-day T_RM_ were generated by i.m. and s.c. immunization.

In subsequent experiments, we examined groups of mice vaccinated by these different routes for their ability to respond to a lethal intranasal challenge of VACV_OVA_. Groups of ten mice assayed 45 days after initial vaccination were subjected to intranasal challenge, and mice were weighed daily after vaccination. Mice that lost >20% of body weight (BW) were killed. Figure 3g, h show that naive mice universally succumbed to the lethal infection, whereas mice immunized s.s. showed minor transient weight loss but complete survival. In contrast, mice vaccinated i.d., s.c., or i.m. lost substantial weight (Fig. 3g), and although 60% of i.d. vaccinated mice survived, only 40% and 30% of mice vaccinated i.m. and s.c., respectively, survived (Fig. 3h). These results are consistent with the superior production of different memory T-cell subsets after vaccination by s.s.

### MVA s.s. generated more than half number of lung T_RM_ compared to intra-tracheal immunization

**Fig. 4:**
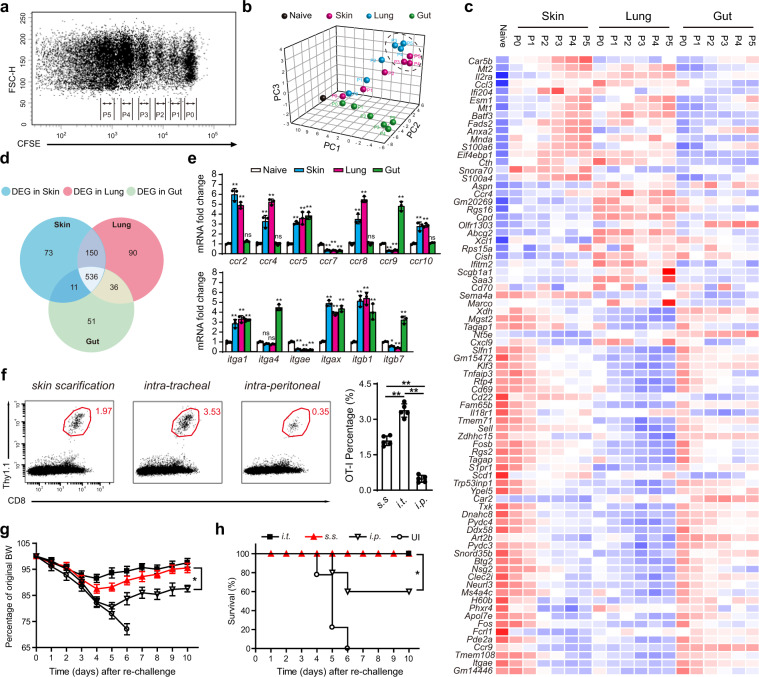
MVA s.s. generates more than half number of lung T_RM_ compared to intra-tracheal (i.t.) and is sufficient to protect mice against lethal respiratory challenge. **a** Flow cytometric analysis of OT-I cell proliferation in draining lymph nodes at 60 h post MVA infection via s.s. CFSE-labeled naive OT-I Thy1.1^+^ cells were transferred into Thy1.2^+^ recipient mice 1 day before mice were infected with 1.8 × 10^6^ pfu MVA-Ova. **b** PCA of gene expression data for 19 CD8^+^ T-cell populations based on CFSE signal and different infection routes. Each dot represents an individual experiment wherein mRNA was pooled from 15 to 20 mice from 3 to 4 independent biological groups (5 mice/group). **c** Heatmap of differentially expressed genes selected from a pairwise comparison between s.s. and intraperitoneal (i.p.) activated T cells. **d** Venn diagram analysis of genes differentially expressed in pairwise comparisons between s.s., i.t., and i.p. activated T cells relative to T_N_ (fold change cutoff, ≥2). **e** qRT-PCR analysis of cell homing molecule gene expression in s.s., i.t., and i.p. activated T cells. **f** Flow cytometric analysis (left) and quantification (right) of lung T_RM_ cells at day 45 post MVA infection via indicated routes. **g**, **h** Body weight (BW) (**g**) and survival measurements (**h**) of WR-VACV re-challenged mice that were immunized previously with MVA via indicated routes 45 days earlier. OT-I WT cells were adoptively transferred into µMT mice before mice were infected with 1.8 × 10^6^ pfu MVA via indicated routes. Forty-five days later, mice were re-challenged with a lethal dose of WR-VACV by intranasal infection. DEG, differentially expressed genes. Graphs show the mean ± SD (*n* = 5). UI = unimmunized. ns = not significant, *p < 0.05, **p < 0.01.

We were struck by the capacity of skin immunization via s.s. to generate both skin T_RM_ and lung T_RM_. Although skin and gut T-cell trafficking have been studied extensively, lung T-cell trafficking has been studied less comprehensively. We immunized CFSE OT-1 loaded mice with MVA_OVA_ via three routes: s.s. to assess skin homing, intraperitoneally (i.p.) to assess gut homing, and intra-tracheally (i.t.) to assess lung homing. At 60 h, T cells were collected from the respective draining LNs (inguinal for skin, mesenteric for gut, and mediastinal for lung) and were sorted based on CFSE expression into cells that had not divided (P0) or had divided once through five times (P1–P5; Fig. 4a). Cells were subjected to transcriptional profiling, and results were analyzed bioinformatically. By PCA analysis, P0 cells from skin, gut, and lung homing nodes clustered near each other (Fig. 4b). However, as early as P1 and clearly by P2, OT-1 cells activated in different nodes diverged significantly in transcriptional profile. In particular, OT-1 cells from mesenteric nodes were quite distinct from OT-1 cells from inguinal and mediastinal nodes (Fig. 4b). Interestingly, P1–P5 cells from inguinal (skin draining) node clustered closely with P1–P5 cells from mediastinal (lung draining) nodes, suggesting similar pathways involved in skin and lung homing imprinting (Fig. 4b). Excluding genes upregulated in all T-cell groups, lung and skin homing T cells shared upregulation of 150 genes, compared to 73 and 90 upregulated in only the skin or only the lung, respectively (Fig. 4c, d). In contrast, only 11 upregulated genes were shared between skin and gut, and only 36 between lung and gut. Examination of genes encoding tissue-homing molecules showed homology between lung and skin immunization (CCR2, CCR4, and CCR10), whereas gut immunization showed unique upregulation of CCR9, α4, and β7 integrins (Fig. 4e). These data suggest a similar pattern of gene expression of T cells activated in skin- vs. lung-draining LN, and a pattern in gut-draining LN that is very different from the lung- and skin-draining LN.

We next directly compared the capacity of skin (s.s.), lung (i.t.), and gut (i.p.) immunization with MVA_OVA_ to generate lung T_RM_. Mice were immunized by the above routes and after 45 days, lung T_RM_ were analyzed. As expected, lung immunization resulted in the highest number of lung T_RM_, but skin immunization by s.s. generated more than half as many T_RM_ in the lung (Fig. 4f). In contrast, i.p. immunization resulted in <10% of the lung T_RM_ generated by lung immunization (Fig. 4f). Similar to the skin T_RM_, lung T_RM_ were CD69^+^, CD103^+^, CD62L^−^, and KLRG1^−^, and expressed E and P-selectin ligands (Supplementary Fig. 4). A companion cohort of mice were subjected to lethal intranasal challenge with VACV_OVA_. Mice immunized i.t. or s.s. showed mild weight loss but 100% recovery and survival (Fig. 4g, h). Mice immunized i.p. showed more severe weight loss, and only 60% survived the infectious challenge (Fig. 4g, h). In another series of experiments, i.t. immunization was compared to s.s. immunization with regard to generation of skin T_RM_. Although s.s. was most efficient at generating skin T_RM_, lung immunization via i.t. generated 50% of the skin T_RM_ compared to s.s. immunization (Supplementary Fig. 5). These data confirm that lung immunization can generate abundant skin T_RM_, and skin immunization can generate abundant lung T_RM_.

## Discussion

Smallpox vaccination via epidermal disruption (also known as s.s.) using VACV provided broad and effective protective immunity against Smallpox caused by Variola major, and led to the eradication of this devastating infectious disease in the twentieth century^14^. MVA is derived from VACV but has lost roughly 10% of the parent genome, including several immune inhibitory genes that block CC chemokines, IFNα/β, IFNγ, tumor necrosis factor-α, and STING^27^, and does not replicate in mammalian cells^20^. In addition to its use as a smallpox vaccine, MVA has been used extensively as a heterologous vaccine vector^15^, although we were unable to find any description of it being delivered through s.s.^21^, except for one study showing that MVA administered by percutaneous inoculation could elicit protective immune responses that are comparable or better to s.c. vaccination^28^. There are no clear reasons that MVA has not been delivered via s.s., other than the assumption that replication was required for this route of administration. Here we show that MVA delivered by s.s. can provoke a potent immune response at doses lower than those used for i.m. and s.c. injection. Dose-sparing effects of MVA after i.d immunization vs. i.m. and s.c. immunization were also found in a human trial using ACAM3000^29^. In a direct comparison of delivery via i.m., s.c., and i.d. routes, s.s. administration of lower doses of MVA provide superior protective immunity against a lethal VACV challenge. These data suggest that similar to VACV, MVA delivered by s.s. provides a potent and durable immune response. We found that both Langerhans cells and CD207^+^ dermal dendritic cells were both required for optimal immunization via this route. In contrast to VACV, mice deficient in adaptive immunity could be safely immunized via s.s. with MVA, supporting the safety of this vector in immunocompromised hosts. One other advantage of the s.s. mode of delivery is dose sparing—doses of MVA that are too low to elicit immune response when given i.m.are highly effective when delivered by s.s.

When used as a heterologous vaccine vector encoding for a T-cell antigen, MVA_OVA_ delivered s.s. provided the earliest and best activation of OVA-specific T cells (OT-1) in draining LNs at 60 h and day 5 post immunization. In contrast, s.c. and i.m. immunization generated the latest and lowest number of activated LN OT-I cells, as indicated by negative CFSE staining (Fig. 2a–d). These results indicated that in addition to a different capacity to generate activated T cells, there are also differences in T-cell activation and proliferation kinetics related to these different antigen delivery routes. Interestingly, CD8^+^ T_eff_ cells in skin-draining LNs at day 5 showed different patterns of gene expression after immunization s.s., i.d., s.c., and i.m., respectively. T cells generated by i.m. were most distinct transcriptionally from those generated by s.s. immunization. When T cells were collected from spleens at day 45 after immunization, cells with T_EM_ markers retained distinct transcriptional profiles, with i.m. immunization-generated T_EM_ cells being most distinct from s.s. immunization-generated T_EM_ cells. Day 45 memory T cells expressing CD62L (T_CM_) showed smaller transcriptional differences between immunization routes, but s.s. generated T_CM_ cells were still readily distinguishable from those generated by i.m. immunization. These surprising data suggest that there are qualitative (e.g., transcriptional) differences in T_eff_ and memory T cells (T_M_) cells generated by immunization route that are evident by day 5 and persist at day 45.

There were also quantitative differences in T_M_ generation depending on route of administration. Immunization via s.s. generated greater numbers of both T_EM_ and T_CM_ at 45 days after immunization. When skin T_RM_ were measured, s.s. generated more CD8^+^ T cells than other routes, with i.m. being least efficient. Because lethal intranasal challenge with VACV results in death from pulmonary inflammation, we also measured lung T_RM_. Accordingly, s.s. generated higher numbers of lung T_RM_ than other routes, consistent with previous reports^11,30^, with i.m. generating fewest lung T_RM_. T_RM_ from skin and lung both expressed CD69 and CD103, with expression of E- and P-selectin ligands detectable as well. When animals were challenged by lethal intranasal infection with VACV_OVA_, mice immunized by s.s. showed minimal weight loss and 100% survival. Mice immunized by all other routes showed greater morbidity and some mortality, with i.m. immunization being least effective. Whether the ability of s.s. immunized mice to universally survive the intranasal challenge of VACV_OVA_ was due to higher numbers of lung T_RM_, circulating T_EM_ and T_CM_, or qualitatively different T_eff_ and memory T cells cannot be determined from these data. However, this suggests that the original method of smallpox vaccination—s.s. administration—appears to be uniquely effective at generating robust protective immunity against airway challenge.

As s.s. immunization was so efficient at generating lung T cells and protective immunity against a pulmonary infectious challenge, we compared skin infection with direct lung infection, and assessed T_eff_ in skin and lung-draining LN, respectively, using i.p. injection and mesenteric nodes as a control. Thus, three routes of immunization were compared—s.s., i.t., and i.p.—and T_eff_ from draining LNs—inguinal, mediastinal, and mesenteric, respectively—were compared by transcriptional profiling. Although proliferating T_eff_ from skin-draining and gut-draining nodes rapidly diverged, proliferating T_eff_ from skin-draining and lung-draining nodes showed significant overlap over time. Notably, α1β1 integrin, CCR4, and CCR8 were preferentially elevated in T cells from skin and lung-draining nodes, and α4β7 and CCR9 were preferentially upregulated in mesenteric LNs, consistent with previously reported data^31–33^. When lung T_RM_ were examined after 45 days, both skin and lung infection generated abundant lung T_RM_, whereas i.p. immunization was less effective at generating these cells. Protection against lethal intranasal challenge was complete in skin and lung immunized mice, but only partial after i.p. immunization. These data suggest that there is substantial overlap in T cells imprinted by skin and lung-draining LNs and suggests that skin immunization is well-suited at generating T cells with lung-tropic properties. Of note, another recent work showed that skin immunization with MVA generated T cells that protected the host against systemic viral spread by seeding distant tissues, including lung^34^. Even though i.t. immunization generated more lung T_RM_ and was equally protective as s.s. immunization, there is concern that using such an approach (e.g., mucosal immunization) in patients might provoke an unacceptable hyperinflammatory response in lung. For this and other reasons, s.s. immunization seems to be a more promising route of administration.

Two important conclusions can be drawn from this study that may be relevant to human disease. First, immunization with MVA generates powerful adaptive immunity, but like VACV the most potent local and systemic adaptive immunity generated occurs after superficial s.s. that involves epidermal disruption. The dose of MVA used in s.s. delivery is lower than required in muscle/i.m. delivery. This suggests the possibility that doses of MVA being stockpiled in anticipation of a dystopian future smallpox attack may protect orders of magnitude for more people if delivered s.s. instead of MVA. The second conclusion is that MVA delivered by s.s. is a very effective way of generating protective CD8^+^ T_M_ in lung, in addition to a more robust circulating T-cell response. MVA vaccines are being developed for respiratory pathogens, including influenza A and respiratory syncytial virus^35,36^, but these are being tested only by i.m. or s.c. injection. Our data strongly suggests that delivering these vaccines via s.s. may generate even more effective protective immunity to pathogens that infect lung. Although there is no direct evidence of correlation between clinical protection efficacy with the findings from this study, it is interesting to speculate about vaccine development against airway viral infections like COVID-19. Future studies using MVA encoding for COVID-19 surface proteins, particularly spike protein, should to be done to assess whether MVA delivered s.s. could provide protective immunity against COVID-19.

There are several limitations to this study, which should be noted. All mouse experiments were performed with CD57Bl/6 strain, primarily to facilitate working with the syngeneic transgenic T-cell line OT-1. Additional strains of mice should be tested to determine whether the superiority of s.s. extends to other strains. And of course, we cannot determine from our data whether s.s. in human subjects would lead to superior T-cell immune response, although i.d. administration of ACAM3000 appears to be more immunogenic than i.m. or s.c.^29^. While the data using the ovalubumin peptide is clear, additional CD8^+^ antigens should ultimately be studied. In addition, although we have shown previously that humoral responses are superior in mice immunized with MVA s.s. vs i.m.^11^, we did not explore humoral responses in the present study. Finally, it will be important to replicate these findings in human subjects, using the currently licensed MVA vaccine.

## Methods

### Mice

WT C57BL/6, CD45.1^+^, Thy1.1^+^, Rag1^−/−^, µMT, Langerin-DTA, and Langerin-DTR mice were purchased from Jackson Laboratory. Thy1.1^+^ Rag1^−/−^ OT-I mice were maintained through routine breeding in the animal facility of Harvard Institute of Medicine, Harvard Medical School. Six- to 8-week-old male mice were used and were randomly assigned to each group before start. All experiments were performed blinded with respect to treatment. For survival experiments, mice that had lost over 25% of original BW were killed.

### Ethics

Animal experiments were performed in accordance with the guidelines put forth by the Center for Animal Resources and Comparative Medicine at Harvard Medical School and were approved by the Harvard/BWH Institutional Animal Care and Use Committee (IACUC).

### Viruses

An attenuated strain (VACV) of WR-VACV was used in some experiments as control vaccine and was a kind gift from Dr. Bernald Moss (National Institutes of Health, Bethesda, MD). WT WR-VACV were purchased from American Tissue Culture Company (ATCC). The virus stocks were expanded and tittered in Hela cells and CV-1 cells (ATCC) by standard procedures. ACAM3000MVA (Acambis MVA) and DF-1 cells were gifted by Dr. Michael Seaman (Beth Israel Deaconess Medical Center, Boston MA). MVA-OVA was gifted by Dr. Ingo Drexler (Technische Universita¨ t Mu¨nchen and Helmholtz Centre Munich, Germany). MVA stocks were expanded and titrated in DF-1 cells.

### Virus infection

Mice were immunized with the MVA or VACV at the indicated doses by s.s.^11^. Briefly, mice were anesthetized with 2,2,2 tribromoethanol (250 mg/kg, Sigma) by i.p. injection with a target of 25–30 min of immobility. Five microliters of trypsinized virus at varying titer were placed on the inoculation skin site, which was then scarified with a 28 g needle (500 μl insulin syringe) by poking 25 times and scratching 25 times, endeavoring to stay within the superficial epidermis and to minimize bleeding. Alternatively, mice were immunized by s.c., i.d., or i.m. injection at the indicated dose. For secondary challenge, memory mice were challenged intranasally with a lethal dose of WR-VACV (2 × 10^6^ pfu in 20 µl of phosphate-buffered saline, PBS) at 6–20 weeks post immunization. The change of BW and survival of mice were monitored daily following challenge for up to 12 days.

### In vitro restimulation assay

Poxvirus-specific T-cell response against poxvirus was assessed at day 7 post challenge. T cells isolated from draining LNs or spleens was suspended in T-cell medium (RPMI containing 10% fetal bovine serum, 2 mM 2-β mercaptoethanol, 1× nonessential amino acid, 1× sodium pyruvate) and were used as effector cells. For target cell preparation, naive splenocytes was infected at 37 °C for 5 h with WR-VACV at a multiplicity of infection of 5 in RPMI medium supplemented with 10% fetal calf serum. After infection, the cells were washed three times with PBS and co-cultured (5 × 10^5^ cells/well) with effector cell at a 1 : 1 ratio in 96-well plate in T-cell medium at 37 °C for 48 h. Uninfected naive splenocytes co-cultured with target cells were used as negative controls. IFN-γ concentration in the culture supernatants were measured by enzyme-linked immunosorbent assay using anti-IFN-γ mAb pairs (BD Pharmingen) according to manufacturer’s protocol.

### Preparation of cell suspensions

LNs and spleens were collected and pressed through a 70 µm nylon cell strainer to prepare cell suspensions. Red blood cells (RBCs) were lysed using RBC lysis buffer (00-4333-57; eBioscience). Skin tissue was excised after hair removal, separated into dorsal and ventral halves, minced, and then incubated in Hanks balanced salt solution supplemented with 1 mg/ml collagenase A (11088785103; Roche) and 40 μg/ml DNase I (10104159001; Roche) at 37 °C for 30 min. After filtration through a 70 μm nylon cell strainer, cells were collected and washed three times with cold PBS before staining. Gate strategy of T cells (Supplementary Fig. 6): T_eff_, CD8^+^ CD90.1^+^ CD44^+^ CD62L^−^; T_CM_, CD8^+^ CD90.1^+^ CD44^+^ CD62L^+^; T_EM_, CD8^+^ CD90.1^+^ CD44^+^ CD62L^−^; T_RM_, CD8^+^ CD90.1^+^ CD44^+^ CD62L^−^ CD69^+^.

### Antibodies and flow cytometry

The following anti-mouse antibodies were obtained from BD PharMingen: PerCP-conjugated anti-CD3e (553067, 1 : 100), PE-conjugated anti-CD8 (557654, 1 : 100), PE-Cy7-conjugated anti-CD8 (552877, 1 : 100), APC-Cy7-conjugated anti-CD8 (557654, 1 : 100), PE-conjugated anti-CD90.1 (561404, 1 : 100), APC-conjugated anti-CD90.1 (557266, 1 : 100), PE-Cy7-conjugated anti-CD62L (560516, 1 : 100), APC-Cy7-conjugated CD62L (560514, 1 : 100), and APC-conjugated anti-IFN-γ (554413, 1 : 100). Biolegend: PE-conjugated anti-CD44 (103008, 1 : 100), PE-Cy7-conjugated anti-CD44 (103030, 1 : 100), PE-Cy7-conjugated anti-CD69 (104512, 1 : 100), and APC-conjugated anti-CD103 (121414, 1 : 100). Flow cytometry data were acquired with a FACS Canto II flow cytometer (BD Biosciences) and data were analyzed with Flowjo software (Tree Star).

### Mouse adoptive transfer and treatment

LNs were collected from naive donor mice at age of 6–8 weeks. T cells were purified by magnetic cell sorting using a mouse CD8α^+^ T-cell isolation kit (130-104-075; Miltenyi Biotec) or a mouse CD4^+^ T-cell isolation kit (130-104-454; Miltenyi Biotec), according to the manufacturer’s protocols. T cells were then transferred intravenously into recipient mice at a total number of 5 × 10^5^. T cells were labeled with CFSE (65-0850; eBioscience) before co-transfer, where indicated.

### Microarray, data analysis, and quantitative real-time PCR

For each group of microarray dataset, OT-I cells from 15 to 20 mice of 3–4 independent biological replicates (5 mice/group) were sorted with a FACSAria III (BD Biosciences) and pooled. RNA was extracted with a RNeasy Micro kit (74004; Qiagen). RNA quality and quantity were assessed with a Bioanalyzer 2100 (Agilent). Then RNA was amplified and converted into cDNA by a linear amplification method with WT-Ovation Pico System (3302-60; Nugen). Subsequently, cDNA was labeled with the Encore Biotin module (4200-60; Nugen) and hybridized to GeneChip MouseGene 2.0 ST chips (Affymetrix) at the Translational Genomics Core of Partners Healthcare, Harvard Medical School. GeneChips were scanned using the Affymetrix GeneChip Scanner 3000 7 G running Affymetrix Gene Command Console version 3.2. The data were analyzed by using Affymetrix Expression Console version 1.3.0.187 using Analysis algorithm RMA. To evaluate overall performance of microarray data, PCA and Pearson’s correlation coefficients among 12 diverse samples were applied by using 26,662 transcripts (R Program). All microarray data has been submitted to the Gene Expression Omnibus (accession code GSE150190).

For relative quantitative real-time PCR, RNA was prepared as described above. Bio-Rad iCycler iQ Real-Time PCR Detection System (Bio-Rad) was used with the following settings: 45 cycles of 15 s of denaturation at 95 °C, and 1 min of primer annealing and elongation at 60 °C. Real-time PCR was performed with 1 μl cDNA plus 12.5 μl of 2 × iQ SYBR Green Supermix (Bio-Rad) and 0.5 μl (10 μM) specific primers. For absolute quantitative real-time PCR, each standard curve was constructed using tenfold serial dilutions of target gene template ranging from 10^7^ to 10^2^ copies per mL and obtained by plotting values of the logarithm of their initial template copy numbers vs. the mean Ct values. The actual copy numbers of target genes were determined by relating the Ct value to a standard curve.

### Determination of viral load

Viral load in various tissues following MVA or VACV s.s. was determined by quantitative real-time PCR. Briefly, DNA was purified using the DNeasy Mini Kit (Qiagen, Valencia, CA). The primers and TagMan probe used in the quantitative PCR assay are specific for the ribonucleotide reductase Vvl4L of VACV. The sequences are: (forward) 5′-GAC ACT CTG GCA GCC GAA AT-3′, (reverse) 5′-CTG GCG GCT AGA ATG GCA TA-3′, (probe) 5′-AGC AGC CAC TTG TAC TAC ACA ACA TCC GGA-3′. The probe was 5′-labeled with FAM and 3′-labeled with TAMRA (Applied Biosystems, Foster City, CA). Real-time PCR was performed with the Bio-Rad iCycler iQTM Real-Time PCR Detection System (Bio-Rad Laboratories). Thermal cycling conditions were 50 °C for 2 min and 95 °C for 10 min for one cycle, followed by 45 cycles of amplification (94 °C for 15 s and 60 °C for 1 min). Standard curve was established from DNA of an MVA or VACV stock with previously determined titer. Corresponding CT values obtained by the real-time PCR reactions were plotted on the standard curve to calculate viral load in the samples. The number of viral DNA copies was normalized to that in the skin samples of uninfected naive mice.

### Statistical analysis

Comparisons for two groups were calculated using Student’s *t*-test (two tailed). Comparisons for more than two groups were calculated with one-way analysis of variance (ANOVA) followed by Bonferroni’s multiple comparison tests. Two-way ANOVA with Holm–Bonferroni post hoc analysis was used to compare weight loss between groups and Log-rank (Mantel–Cox) test was used for survival curves. *p* < 0.05 was considered statistically significant.

### Reporting summary

Further information on research design is available in the Nature Research Reporting Summary linked to this article.

## Supplementary information

Supplementary Information

Reporting Summary

## Data Availability

Microarray data have been submitted to the Gene Expression Omnibus (accession code GSE150190).
