# Supplementary material for: Epicutaneous immunization with modified vaccinia Ankara viral vectors generates superior T cell immunity against a respiratory viral challenge
Source: NPJ Vaccines. 2021 Jan 4;6:1. doi: 10.1038/s41541-020-00265-5 (PMC7782760; doi:10.1038/s41541-020-00265-5)
Supplement: Supplementary file 1 — Supplementary Information [file 41541_2020_265_MOESM1_ESM.pdf]

Supplementary Figure Legends

Supplementary Figure 1. MVA skin scarification induced smaller pox lesions that healed significantly faster compared to VACV skin scarification in immunocompetent mice. C57BL/6 mice were immunized with 1.8 X 10^6 pfu MVA or VACV by skin scarification. Photographs of pox lesion were taken on day 4, 7, 14 and 28 post-immunization.

Supplementary Figure 2. Delivery of MVA via s.s. generates stronger cellular responses compared to i.d., s.c., and i.m. infection routes. C57BL/6 mice were immunized with 1.8 X 10^6 pfu MVA via indicated routes. Activated T cells in draining lymph nodes (a) and spleen (b) were isolated at 7 days post infection, and T cell response against VACV was measured based on IFN-γ secretion. Symbols represent individual mice (n = 5 mice/group). $*p < 0.05$, $**p < 0.01$.

Supplementary Figure 3. Delivery of MVA via s.s. generates T cells that are qualitatively distinct from those generated from i.d., s.c., i.m.. a-b. Venn diagram analysis of genes up-regulated (a) or down-regulated (b) in pairwise comparisons between T cells activated via MVA s.s., i.d., s.c., i.m. (day 5) relative to that of T_N. **c-d.** Fold change analysis of genes shared among s.s., i.d., s.c. and i.m. activated T cells (day 5) relative to that of T_N. c, 146 shared up-regulated genes, d, 41 shared down-regulated genes. **e.** qRT-PCR analysis of cell homing molecule gene expression in s.s., i.d., s.c. and i.m. activated T cells (day 5) relative to that of T_N. Graphs show the mean ± s. d. (n=5). ns = not significant, $*p < 0.05$, $**p < 0.01$.

Supplementary Figure 4. Phenotyping of tissue-resident memory T cell surface marker on lung $CD8^+$ T_{RM} cells generated by MVA infection via skin scarification, intra-tracheal administration or intra-peritoneal injection. Flow cytometric analysis of T cell proliferation and homing receptor expression on OT-I cells residing in lung at 45 days post MVA infection.

Naïve OT-I Thy1.1$^+$ cells were transferred into Thy1.2$^+$ recipient mice one day before mice were infected with 1.8×10^6 pfu MVA-Ova by s.s., i.t. or i.p.. At 45 days after infection, proliferation and tissue-homing receptor expression of OT-I T_{RM} cells isolated from lung tissue were analyzed by flow cytometry. Data are representative of three independent experiments (n = 5 mice per group). ESL, E-selectin ligand.

Supplementary Figure 5. Skin T_{RM} cells generated by MVA infection via skin scarification, intra-tracheal administration or intra-peritoneal injection. Flow cytometric analysis and quantification of skin T_{RM} cells at day 45 post 1.8×10^6 pfu MVA infection via indicated routes. Data are representative of three independent experiments (n = 5 mice per group). Graphs show the mean ± s. d. (n=5). $**p < 0.01$.

Supplementary Figure 6. Gating strategy for the analysis of memory OT-I cell populations.

Supplementary Table 1. List of abbreviation.

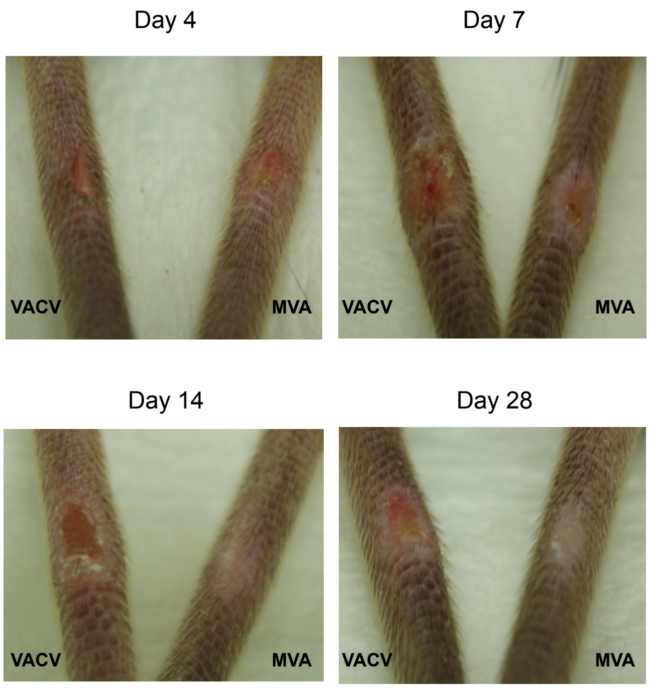

Supplementary Figure 1

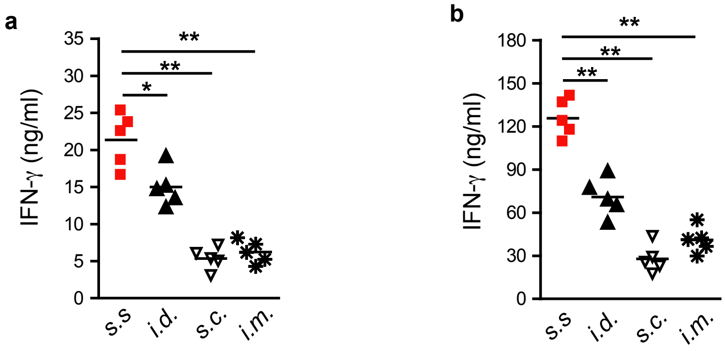

Supplementary Figure 2

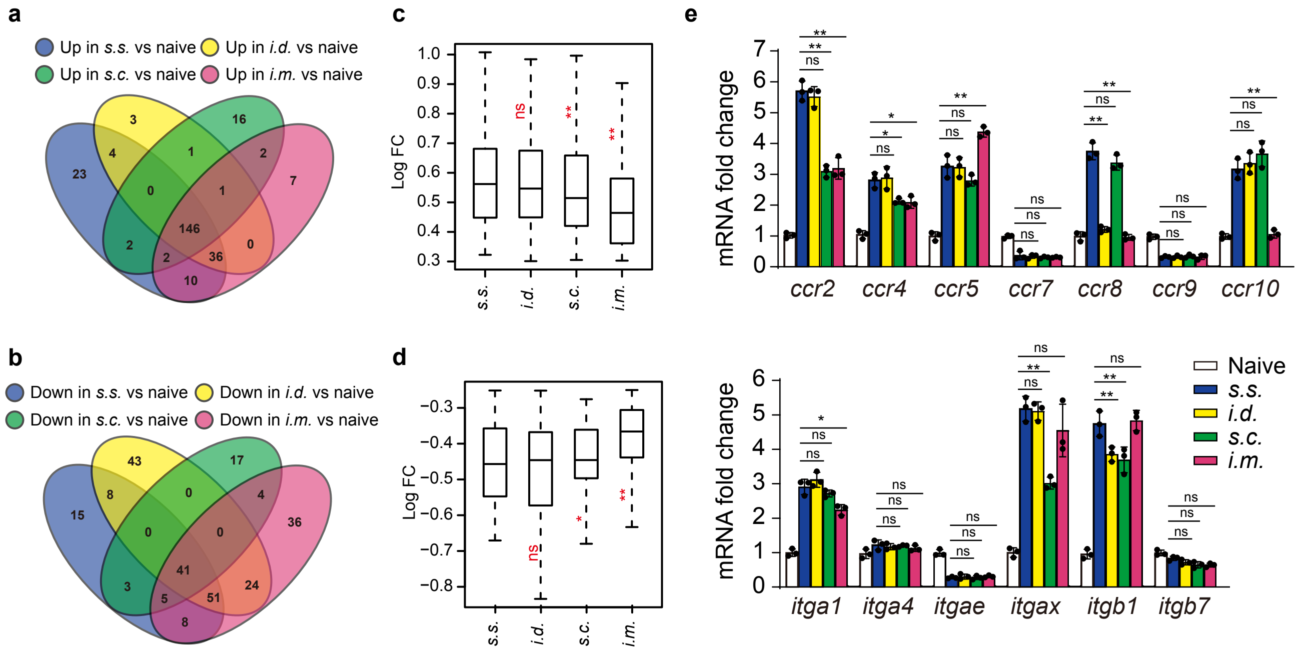

Supplementary Figure 3

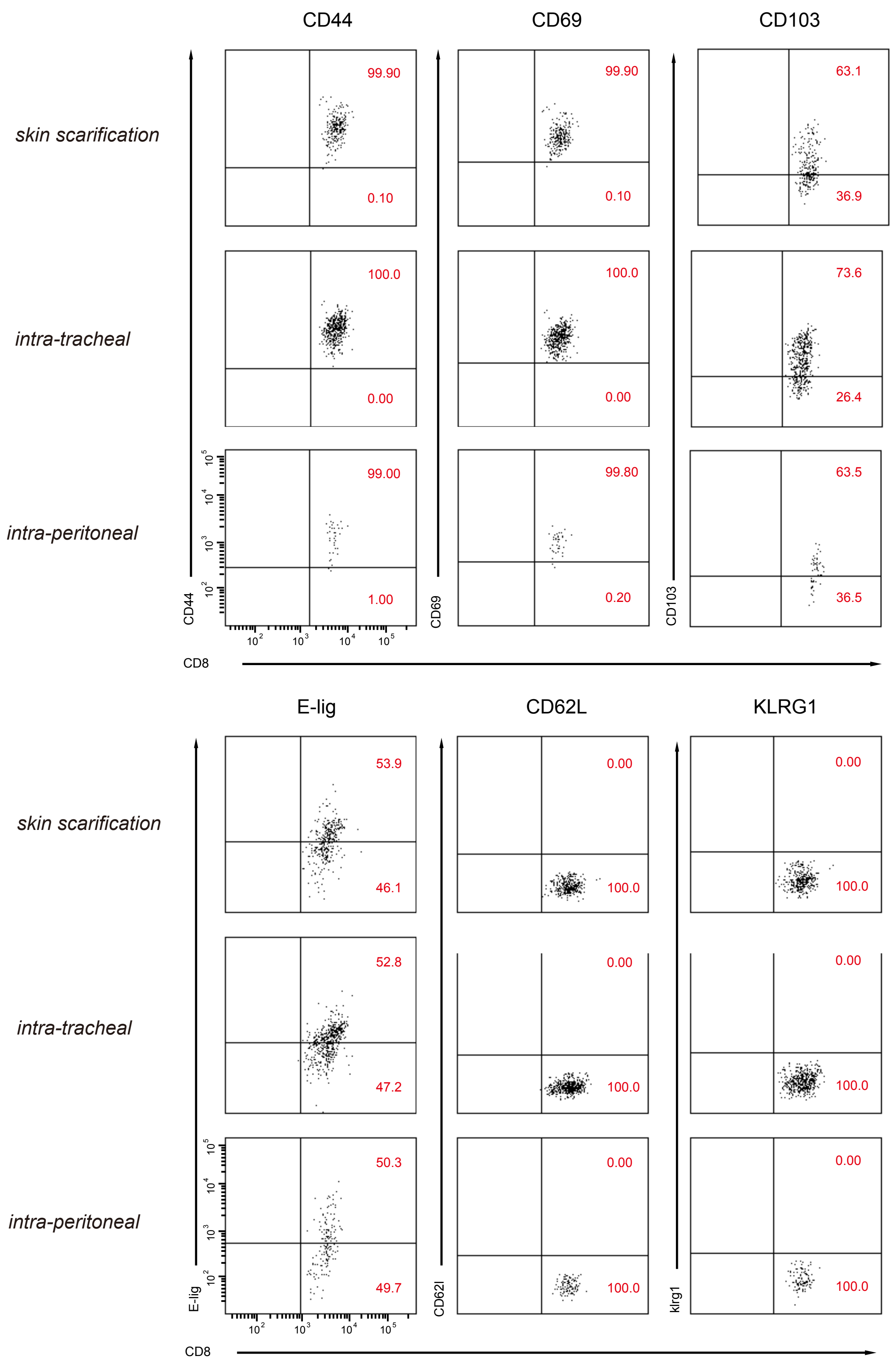

Supplementary Figure 4

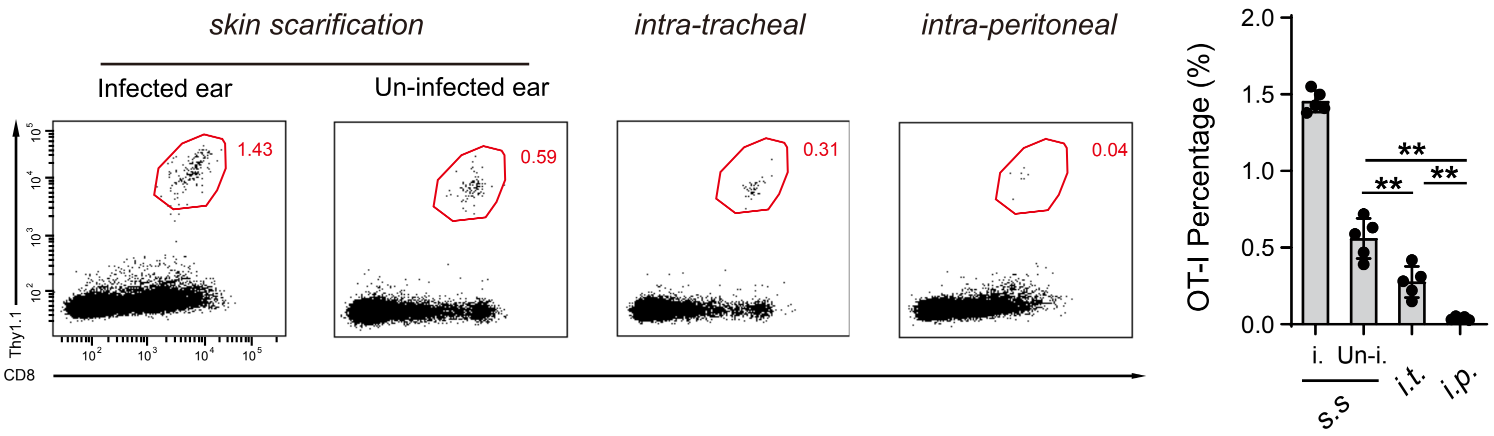

Supplementary Figure 5

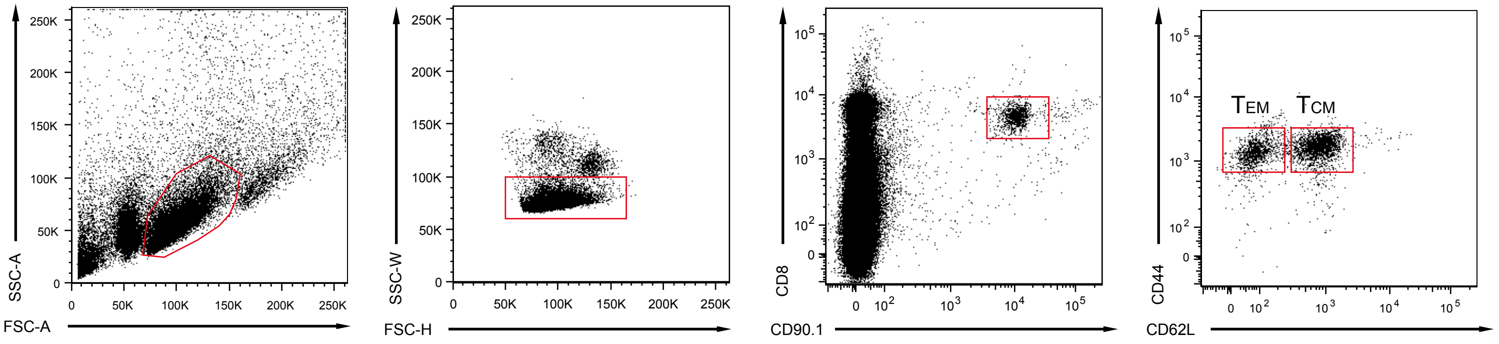

Supplementary Figure 6

Abbreviation	Definition
MVA	Modified Ankara Virus
VACV	Vaccinia Virus
OVA	Ovalbumin
WR-VACV	Western-reserve Vaccinia Virus
s.s.	skin scarification
i.d.	intradermal
s.c.	subcutaneous
i.m.	intramuscular
i.t.	intratracheal
i.p.	intraperitoneal
T_{eff}	Effector T cells
T_{RM}	Tissue resident memory T cells
T_{CM}	Central Memory T cells
T_{EM}	Effector Memory T cells
LN	lymph nodes
PCA	Principle Component Analysis
WT	wide-type
BW	Body Weight
qRT-PCR	Quantitative real time PCR

Supplementary Table 1
