# Supplementary material for: Epicutaneous immunization with modified vaccinia Ankara viral vectors generates superior T cell immunity against a respiratory viral challenge
Source: NPJ Vaccines. 2021 Jan 4;6:1. doi: 10.1038/s41541-020-00265-5 (PMC7782760; doi:10.1038/s41541-020-00265-5)
Supplement: Supplementary file 2 — Reporting Summary [file 41541_2020_265_MOESM2_ESM.pdf]

nature research

Corresponding author(s): Thomas S. Kupper, M.D.

Last updated by author(s): Sep 21, 2020

Reporting Summary

Nature Research wishes to improve the reproducibility of the work that we publish. This form provides structure for consistency and transparency in reporting. For further information on Nature Research policies, see Authors & Referees and the Editorial Policy Checklist.

Statistics

For all statistical analyses, confirm that the following items are present in the figure legend, table legend, main text, or Methods section.

n/a	Confirmed	
☐	☒	The exact sample size (*n*) for each experimental group/condition, given as a discrete number and unit of measurement
☐	☒	A statement on whether measurements were taken from distinct samples or whether the same sample was measured repeatedly
☐	☒	The statistical test(s) used AND whether they are one- or two-sided<br>*Only common tests should be described solely by name; describe more complex techniques in the Methods section.*
☐	☒	A description of all covariates tested
☐	☒	A description of any assumptions or corrections, such as tests of normality and adjustment for multiple comparisons
☐	☒	A full description of the statistical parameters including central tendency (e.g. means) or other basic estimates (e.g. regression coefficient) AND variation (e.g. standard deviation) or associated estimates of uncertainty (e.g. confidence intervals)
☒	☐	For null hypothesis testing, the test statistic (e.g. F, t, r) with confidence intervals, effect sizes, degrees of freedom and P value noted<br>*Give P values as exact values whenever suitable.*
☒	☐	For Bayesian analysis, information on the choice of priors and Markov chain Monte Carlo settings
☒	☐	For hierarchical and complex designs, identification of the appropriate level for tests and full reporting of outcomes
☒	☐	Estimates of effect sizes (e.g. Cohen's d, Pearson's r), indicating how they were calculated

Our web collection on statistics for biologists contains articles on many of the points above.

Software and code

Policy information about availability of computer code

Data collection	N/A
Data analysis	N/A

For manuscripts utilizing custom algorithms or software that are central to the research but not yet described in published literature, software must be made available to editors/reviewers. We strongly encourage code deposition in a community repository (e.g. GitHub). See the Nature Research guidelines for submitting code & software for further information.

Data

Policy information about availability of data

All manuscripts must include a data availability statement. This statement should provide the following information, where applicable:

- Accession codes, unique identifiers, or web links for publicly available datasets
- A list of figures that have associated raw data
- A description of any restrictions on data availability

Microarray data has been submitted to the Gene Expression Omnibus (accession code GSE150190)

Field-specific reporting

Please select the one below that is the best fit for your research. If you are not sure, read the appropriate sections before making your selection.

☒ Life sciences ☐ Behavioural & social sciences ☐ Ecological, evolutionary & environmental sciences

For a reference copy of the document with all sections, see nature.com/documents/nr-reporting-summary-flat.pdf

Life sciences study design

All studies must disclose on these points even when the disclosure is negative.

Sample size	6-8-week-old male mice were used and were randomly assigned to each group (5 mice per group) before start. All experiments were performed blinded with respect to treatment.
Data exclusions	No data were excluded.
Replication	All experiments were repeated at least x 3.
Randomization	Mice were randomly assigned to each group.
Blinding	All experiments were performed blinded with respect to treatment.

Reporting for specific materials, systems and methods

We require information from authors about some types of materials, experimental systems and methods used in many studies. Here, indicate whether each material, system or method listed is relevant to your study. If you are not sure if a list item applies to your research, read the appropriate section before selecting a response.

Materials & experimental systems

n/a	Involved in the study	
☐	☒	Antibodies
☒	☐	Eukaryotic cell lines
☒	☐	Palaeontology
☐	☒	Animals and other organisms
☒	☐	Human research participants
☒	☐	Clinical data

Methods

n/a	Involved in the study	
☒	☐	ChIP-seq
☐	☒	Flow cytometry
☒	☐	MRI-based neuroimaging

Antibodies

Antibodies used	Please see Materials and Methods
Validation	All antibodies were validated per details on the manufacturers website.

Animals and other organisms

Policy information about studies involving animals; ARRIVE guidelines recommended for reporting animal research

Laboratory animals	Wide-type (WT) C57BL/6, CD45.1+, Thy1.1+, Rag1−/−, µMT, Langerin-DTA, Langerin-DTR mice were purchased from Jackson Laboratory. Thy1.1+ Rag1−/− OT-I mice were maintained through routine breeding in the animal facility of Harvard Institute of Medicine, Harvard Medical School.
Wild animals	N/A
Field-collected samples	N/A
Ethics oversight	Animal experiments were performed in accordance with the guidelines put forth by the Center for Animal Resources and Comparative Medicine at Harvard Medical School and were approved by the Harvard/BWH IACUC.

Note that full information on the approval of the study protocol must also be provided in the manuscript.

Flow Cytometry

Plots

Confirm that:

☒ The axis labels state the marker and fluorochrome used (e.g. CD4-FITC).

☒ The axis scales are clearly visible. Include numbers along axes only for bottom left plot of group (a 'group' is an analysis of identical markers).

☒ All plots are contour plots with outliers or pseudocolor plots.

☒ A numerical value for number of cells or percentage (with statistics) is provided.

Methodology

Sample preparation	Lymph nodes and spleens were harvested and pressed through a 70-µm nylon cell strainer to prepare cell suspensions. Red blood cells were lysed using RBC lysis buffer (00-4333-57; eBioscience). Skin tissue was excised after hair removal, separated into dorsal and ventral halves, minced, and then incubated in Hanks balanced salt solution (HBSS) supplemented with 1mg/ml collagenase A (11088785103; Roche) and 40µg/ml DNase I (10104159001; Roche) at 37°C for 30min. After filtration through a 70-µm nylon cell strainer, cells were collected and washed three times with cold PBS before staining.
Instrument	BD FACS Canto II Flow Cytometer
Software	BD FACS Canto II Analyzer
Cell population abundance	Labeled on the figures.
Gating strategy	Gate strategy of T cells (Extended data, Fig. 6): Teff, CD8+ CD90.1+ CD44+ CD62L-; TCM, CD8+ CD90.1+ CD44+ CD62L+; TEM, CD8+ CD90.1+ CD44+ CD62L-; TRM, CD8+ CD90.1+ CD44+ CD62L- CD69+.

☒ Tick this box to confirm that a figure exemplifying the gating strategy is provided in the Supplementary Information.
